# A model for pediatric and neuropsychological screening assessment of children with learning disabilities

**DOI:** 10.1590/S1980-57642012DN06010004

**Published:** 2012

**Authors:** Claudia Berlim de Mello, Leila Raquel Russowsky Brunoni, Ana Luiza Pilla, José Augusto Aguiar Carrazedo Taddei, Thais Barbosa, Elaine Girão Sinnes, Camila Cruz Rodrigues, Monica Carolina Miranda, Mauro Muzskat, Orlando Francisco Amodeo Bueno

**Affiliations:** 1 Doutora, Núcleo de Atendimento Neuropsicológico Infantil, Centro Paulista de Neuropsicologia/AFIP.; 2 Médica, Centro de Genética Médica, Universidade Federal de São Paulo (UNIFESP), São Paulo SP, Brazil.; 3 Médica, Centro de Genética Médica, UNIFESP.; 4 Professor Associado, Disciplina de Nutrologia do Departamento de Pediatria, UNIFESP.; 5 Doutora, Núcleo de Atendimento Neuropsicológico Infantil, Centro Paulista de Neuropsicologia/AFIP.; 6 Mestranda, Departamento de Pediatria, UNIFESP.; 7 Doutora, Núcleo de Atendimento Neuropsicológico Infantil, Centro Paulista de Neuropsicologia/AFIP.; 8 Doutora, Núcleo de Atendimento Neuropsicológico Infantil, Centro Paulista de Neuropsicologia/AFIP.; 9 Doutor, Núcleo de Atendimento Neuropsicológico Infantil, Centro Paulista de Neuropsicologia/AFIP.; 10 Professor Livre Docente, Departamento de Psicobiologia, UNIFESP.

**Keywords:** neuropsychological screening, interdisciplinary assessment, children, primary care, DSM-IV

## Abstract

**Objectives:**

The high frequency of learning difficulties, attention disorders or developmental delay in children in the early years of schooling has resulted in a greater demand for pediatric services. Such services generally include assessments covering various specialties, are lengthy and often inaccessible to families due to prohibitively high cost. This paper presents an economically efficient model of interdisciplinary diagnosis.

**Methods:**

A group of 109 Brazilian students from public schools aged between 5 and 14 years old, referred by teachers for a history of learning disabilities, behavioral changes or language problems, was evaluated at the NANI (Nucleo de Atendimento Neuropsicologico Infantil). Assessments were performed simultaneously during a single day's attendance and comprised clinical-genetic examination, behavioral assessment and neuropsychological screening, specially developed for the process. The multiaxial system of DSM-IV was adopted for diagnostic description.

**Results:**

The results revealed heterogeneity in diagnoses which included specific learning disorders (25.7%), mild intellectual disabilities (17.43%), as well as suspected dysmorphic features (11.93%). Logistic regression showed good sensitivity of neuropsychological screening in the detection of predictive factors for specific developmental disorders, while working memory (p=0.05) and language (p=0.02) problems were found to be higher risk.

**Conclusions:**

The model adopted proved to be useful for defining the diagnosis of several conditions in infancy, and can be incorporated into specialized clinics such as psychiatric or developmental pediatric services.

## Abstract (pt)

**Objetivos:**

A alta frequência de dificuldades de aprendizagem, distúrbios atencionais ou atraso no desenvolvimento cognitivo, em crianças nos primeiros anos de escolaridade, vem acarretando grande demanda por serviços de saúde. Tais serviços em geral abrangem avaliações em diversas especialidades, realizadas em períodos de tempo prolongados, frequentemente de pouca acessibilidade para as famílias devido ao alto custo. Neste trabalho apresenta-se um modelo de diagnóstico interdisciplinar economicamente viável.

**Métodos:**

Foram avaliados os dados de um grupo de 109 alunos da rede pública de ensino entre 5 e 14 anos de idade, encaminhadas ao NANI (Nucleo de Atendimento Neuropsicológico Infantil) pelas professoras por apresentarem história de dificuldades de aprendizagem, alterações comportamentais ou problemas de linguagem oral. As avaliações foram realizadas conjuntamente em um único dia de atendimento e abrangeram exame clínico-genético, avaliação comportamental e uma triagem neuropsicológica qualitativa, especialmente desenvolvida para o processo. Adotou-se o sistema multiaxial do DSM-IV na descrição diagnóstica.

**Resultados:**

Os resultados revelaram uma heterogeneidade de diagnósticos, incluindo transtornos específicos de aprendizagem (25,7%), deficiência intelectual leve (17,43%) e presença de dismorfias a esclarecer (11,93%). Análises de regressão logística evidenciaram boa sensibilidade da triagem neuropsicológica na detecção de fatores preditivos para transtornos específicos do desenvolvimento, sendo que problemas de memória operacional (p=0,05) e de linguagem (p=0.02) se evidenciaram como de maior risco.

**Conclusões:**

O modelo adotado mostrou-se útil, assim, na delimitação diagnóstica de queixas de diversas condições na infância, podendo ser incorporado em clínicas especializadas tais como psiquiátricas ou pediátricas.

## INTRODUCTION

Complaints of learning difficulties, attention disorders, or developmental delay constitute one of the most frequent reasons for referral of children to paediatric neurologists, psychologists or speech and language therapists. In most cases, referrals occur in the first few years of elementary school, when problems during the literacy process arise or when children fail to reach the expected levels of academic achievement. The impact that such difficulties can have on the child's development creates the need for accurate diagnosis, identification of specific educational demands, and family support. At the same time, the multiple factors that may contribute to the onset of learning difficulties or behavioural problems, including environmental (socio-economic, family-related) and neurobiological (clinical, genetic) factors, also call for coordinated actions by healthcare and educational teams. In summary, failing in school raises questions concerning the integration of multidisciplinary teams and the efficiency of assessment models for reaching a dynamic diagnosis and planning early intervention strategies.

The diagnosis based on poor academic performance involves, primarily, the distinction between learning difficulties and actual disabilities.^1-3^ Learning difficulties may be caused by educational inadequacy, circumstantial family-related factors, socio-economic or affective-emotional problems, or can be secondary to sensorial alterations, psychiatric disorders, intellectual deficiency, and chronic or neurological diseases. Learning disabilities, however, according to the DSM-IV definition,^1^ are *diagnosed when the individual's achievement on individually administered, standardized tests in reading, mathematics, or written expression is substantially below that expected for age, schooling, and level of intelligence.* Therefore, learning disabilities refer to lack of specific reading, writing and mathematic abilities which are not compatible with the individual's developmental level, intellectual capacity and schooling level, as a result of intrinsic constitutional factors, probably of neurobiological origin.^3,4^ In Brazil, epidemiological data indicate that 30% to 40% of children in early schooling years have some type of learning difficulty, and 3% to 5% present disabilities.^2^ Other authors hold that the prevalence of learning disabilities affects 5% to 10%, reaching levels of up to 17%.^5^

According to these distinctions, the clinical diagnosis for learning disabilities must be based on the results of intellectual and neuropsychological assessments, as well as on psychosocial and academic performance investigations. Additionally, neurological problems such as chronic non-progressive encephalopathy in children and genetic syndromes should be excluded. Possible comorbidities with other conditions, such as Attention Deficit Hyperactivity Disorder (ADHD), Oppositional Defiant Disorder and Conduct Disorder must also be considered.

Therefore, diagnostic interdisciplinary models are especially indicated for a global comprehension of learning difficulties, essential for the planning of intervention programmes in educational and health contexts. A neuropsychological approach in such models is also particularly important, as it involves the analysis of children's performance in complex cognitive functions such as perception, attention, memory and language. The results of analyses enable the identification of learning difficulty subtypes associated with specific clinical, neuropsychological or psychosocial profiles, and can therefore contribute to the definition of more refined educational interventions. Also, questions concerning familial, educational and social systems should also be considered in the process of diagnostic investigation.

In this sense, the adoption of the DSM-IV Multiaxial System seems particularly promising. It is a model for clinical diagnosis, which consists of axes that are each associated with different information domains, including psychosocial and environmental problems. Axis 1 describes actual clinical disorders, such as specific learning disorders; Axis 2 describes intellectual deficiency; Axis 3 comprises medical conditions associated with the main diagnosis, and Axis 4 describes psychosocial and environmental problems, such as those related to socio-economic conditions, which may interfere in the evolution of the disease.

Therefore, the adequate diagnosis of learning disorders depends on complex investigation procedures and on the involvement of a specialised professional team. Generally, assessment procedures such as psychodiagnosis or neuropsychological examination are lengthy and complex, and often become inaccessible to the low income population. Due to the high financial cost that it entails, the feasibility of traditional neuropsychological assessment in health units, with several individual meetings and the application of various tests and procedures, has been questioned by some researchers from the health care field, who emphasise the importance of an economically efficient evaluation model- a challenge for health units around the world.^6^

In Brazil, neuropsychological screening procedures have been developed aimed at speeding up diagnostic and intervention processes for patients with complaints of cognitive dysfunctions, such as the Mini-Mental Examination^7^ and Neupsilin for adults.^8^ For children, however, there are very few instruments of this nature available, especially those incorporating an interdisciplinary approach.

The present study discusses the results of an interdisciplinary diagnostic investigation conducted in children with complaints of learning difficulties, from 2005 through 2007, who were referred for assessment at the Núcleo de Atendimento Neuropsicológico Infantil (NANI). The assessment involved a multidisciplinary team of health professionals, child neurologists, neuropsychologists, speech and language therapists, paediatricians and clinical geneticists.

Multidisciplinary assessment protocols, including clinical genetic examination and qualitative neuropsychological screening, were especially created for use in these children. The aim was to put together a screening model that simultaneously encompassed the needs of the children and families and also those of the teachers, seeking optimisation through an integrated team effort in the diagnostic process, and that could also be incorporated at a later stage into the public educational and health network.

## METHODS

**Casuistic.** The multidisciplinary diagnostic investigation involved 109 children, predominantly males (66%), attending one of the first four years of Elementary School in one of São Paulo City's municipalities. The mean age of children was 8 years and 7 months (SD 2.9). The children were previously referred by their teachers because of several complaints such as:

(1) persistent difficulties reading and writing, not remedied by conventional educational actions;
(2) presence of substantial social interaction difficulties or dysfunctional behaviour, such as negativity and aggressiveness, affecting the learning process; and
(3) considerable delay in speech development.

Therefore, the children were identified within the municipality's educational network, which comprises a population of around one million students in Elementary School (Education Secretariat of the Municipality of São Paulo; www.sp.gov.br accessed in 23/09/2009).

**Procedures.** Groups of four children along with their parents were referred to the unit for a day of screening. Hence, all procedures included in the interdisciplinary diagnostic assessment were performed during one day spent at the unit by each subject and his/her parent, over a period of four hours with a lunch break. The procedures included appointments for clinical genetic testing and neuropsychological screening. Education coordinators of each participating school also followed the groups.

At the first individual appointment with the professionals involved in the research, all procedures associated with the diagnostic investigation were explained to parents or main caregiver. Information concerning the identification of the child's main caregiver (e.g., biological mother, grandmother, step mother) and respective schooling level (in years) was registered, with the objective of understanding family conditions. The parent or main caregiver signed an informed consent agreement authorising the child's participation in the assessments.

Subsequently, an interdisciplinary assessment of the children was performed by a team of about seven professionals from NANI (all experts in the adopted procedures) followed by meetings for analysis of results. The referred procedures were namely anamnesis and clinical genetic testing, qualitative neuropsychological screening and finally, a behavioural investigation, amounting to 3.5 hours of overall assessment per child. All procedures are described in detail below.

The team met on a daily basis at the end of the procedures in order to discuss diagnostic hypotheses for the four children seen on the day, based on DSM-IV's criteria and the multiaxial system. These meetings lasted about two hours. At a later date, further meetings with professionals (educational coordinators and teachers) from the schools were held in order to present results and put forward suggestions of interventions pertinent to each case.

*Anamnesis and clinical genetic testing -*In an appointment with one of the two paediatricians, a pre-tested and pre-coded questionnaire was used to collect information from the caregiver through anamnesis inquiring about previous and current clinical history, neuropsychomotor development, as well as gestational, perinatal and family history (blood relation of parents, use of alcohol or drugs during pregnancy and child's birth conditions). Low birth weight (<2500g) and length of stay in hospital longer than three days (indicator of birth complications) were recorded since they constitute developmental risk factors. The neuropsychomotor development investigation involved a questionnaire on gait and speech acquisition, based on the Denver II development scale adapted for the Brazilian population.^9^

The anamnesis was complemented by an inquiry into associated medical conditions (Axis 3 in DSM-IV multiaxial system) including epilepsy, previous and current diseases, and sensorial and motor deficiencies.

After the anamnesis, clinical genetic testing including anthropometric measurements (weight, height and cephalic perimeter) and physical examination was performed by a clinical genetician, paying special attention to the presence of mild or severe dysmorphisms, aiming to identify phenotypes that suggested presence of a genetic syndrome. Additionally, the presence of signs of chronic non-progressive encephalopathy, such as gait alterations or spasticity, was investigated. The instrument for anamnesis data collection and clinical genetic testing was specifically developed for this purpose.

*Neuropsychological screening -*Initially, a global intellectual development assessment using Raven's Colored Matrices was carried out. For children older than 12 years, an estimated IQ based on two tests from the WISC-III scale (Cubes and Vocabulary) was used. Subsequently, the simplified neuropsychological assessment (screening), composed of qualitative and quantitative tests based on traditional neuropsychological tests was conducted.^10,11^

The qualitative neuropsychological screening was organised taking into consideration the child's performance in specific functions. The selected tasks could be quickly applied indistinctly by the health professionals for an initial screening result, thus minimising the examiner's subjective perception of the child's general condition and cognitive performance.

The performance on each of the tasks was assessed qualitatively being scored as (0) when the child did not perform any of the task items adequately; (1) when the child executed at least one of the task items adequately; (2) when all the items were executed adequately, as expected for the particular age group. The skills investigated are described below.

- Self care: Aspects related to self-care were assessed in terms of the child's independence for dressing, eating and hygiene. At ages of between 5 and 7 years, the child's performance was considered adequate even when there was a need for parental supervision in these areas. From this age onwards, complete independence was expected.
- Drawings: The children were asked to perform free drawing, and performance assessed according to the evolution of the drawings produced.^12^ Hence, the presence of recognisable figures in the investigated sample, which involved the 5 to 14 year age group, was expected from 5 years and up whereas complete scenes were expected from 7 years of age.
- Working memory: The investigation on verbal working memory was based on oral repetition of sequences of 2 and 3 digits, initially in direct order and then in reverse order.
  For the investigation of visual spatial working memory, a task consisting of a page with 5 randomly distributed blue squares against a white background was utilised. The child was asked to point at a sequence of 2 and 3 squares initially presented by the examiner, first in direct order and then in reverse order. An additional page with numbers was used in order to help the examiner point at the sequences (Appendix 1).
- Visual constructive skills: The visual constructive skills task consisted of copying four simple shapes (a T-shape, a circle, a cross and a rhombus), with performance assessed based on the Child Neurological Examination.^13^ Based on these parameters, the adequate reproduction of the first three figures is expected from children aged 5 to 6 years. The adequate reproduction of the rhombus is expected from children from the age of 7 upwards.
- Visual selective attention: Visual selective attention skills were investigated by asking the children to find 6 target figures - representing familiar objects - in succession. The targets were displayed among 50 distracting figures, randomly distributed on a page with a white background, and the maximum time allowed for their selection was 5 minutes.
- Language: Speech (articulation), verbal expression and verbal comprehension skills were examined in the language assessment. Aspects of speech and verbal expression skills were analysed based on observations of speech speed (normal, slow, accelerated); temporal logical speech sequence, and sentence structure (adequate; alterations in sentence structure, such as agrammatical sentences). The investigation of comprehension skills included analysis of children's answers to questions presented by the examiner after the oral presentation of a short story. For the assessment of expressive skills, children were asked to tell a story based on the free drawings. Pragmatic aspects were investigated based on observations of the child's performance during the dialogue (visual contact; gestural communication; spontaneous participation and respec

*Behavioural assessment -*The assessment of behavioural issues was based on the Child Behavior Checklist - CBCL.^14^ For this assessment, the presence of behavioural problems related to symptoms indicative of depression, anxiety, oppositional defiant disorder, conduct disorder and attention deficit disorder at a clinical level, contributed to the diagnosis of disorders diagnosed for the first time during childhood (DSM-IV Axis 1).

**Statistical analysis.** Logistic regression models were adjusted using the Backward Stepwise Wald method.^15^ The dependent set of variables consisted of each of the two diagnostic axes expressed only by the positive (1) or negative (2) diagnostic criterion. The independent set consisted of six measures (domains) derived from the neuropsychological screening: self-care, drawings, working memory, visual construction, attention and language. The measures (or domains) were also stratified in a way that segmented the sample into either altered scores (lower than 2 in each of the six domains) or normal scores (higher than 2 in each of the six domains). Based on the stratified data, odds ratios were calculated for each of the six domains, according to the presence or absence of positive diagnosis, for each of the axes independently. The independent variables that had significant association with each of the axes separately were verified.

Finally, the data concerning the percentage of right answers for each axis based solely on the significant independent variables and with the presence of all independent variables (canonical correlation) is shown. Also, the model adequacy indexes for each of the axes were calculated.^16^ The level of significance adopted was 5% and the software programme utilised in statistical treatment was SPSS 13.0.

## RESULTS

The assessment of family conditions indicated that the main caregivers in the investigated sample were predominantly biological parents or the mother alone. More than half of the caregivers reported being either illiterate or not having concluded Primary School education (Table 1).

**Table 1:** Demographic variables of the 109 children assessed.

		N	%
**Gender**	Female	36	33.03
	Male	73	66.97
**Main caregivers**	Father and mother (biological)	40	36.70
	Mother alone	36	33.03
	Grandmother	4	3.67
	Other conditions	23	21.10
**Education of caregivers**	Illiterate	7	6.42
	Elementary school (not completed)	54	49.54
	Elementary school (completed)	9	8.26
	High school (not completed)	4	3.67
	High school (completed)	13	11.93

After the application of scales and diagnostic assessments, the participants discussed possible diagnoses based on the four axes contained in the DSM-IV (Table 2).

**Table 2:** Distribution of diagnoses according to DSM-IV Axes.

		N	%
**Axis I**	No diagnosis or condition on Axis I	55	50.46
	Learning disorder NOS	14	12.84
	Disorder of written expression	14	12.84
	Attention-deficit/Hyperactivity disorder	4	3.67
	Conduct disorder	10	9.17
	Oppositional defiant disorder	3	2.75
	Pervasive developmental disorder	9	8.26
**Axis II**	Normal intellectual functioning	44	40.37
	Borderline intellectual functioning	14	12.84
	Mild intellectual disability	19	17.43
	Intellectual disability, severity unspecified	28	25.69
**Axis III**	No diagnosis or condition on Axis III	82	75.23
	Presence of dysmorphic features (to clarify)	13	11.93
	Dysmorphic features (to clarify) with macrosomia	5	4.59
	Definitive diagnosis	9	8.26
**Axis IV**	No problems reported on Axis IV	64	58.72
	Isolated problems	31	28.44
	Multiple problems	14	12.84

Table 3 shows that the variables "working memory" and "language" were significant predictors of positive diagnosis on Axis 1. This means the diagnosis for specific developmental disorders in early childhood was associated with impairment in these tasks. Results indicated that, of children with positive diagnosis on Axis 1, 77.8% showed working memory problems, while 55.6% showed language problems. Children with working memory deficits appeared to be 5.3 times, and those with language delay 26.7 times, more likely to present a positive diagnosis on Axis 1 in comparison to children without these problems. The confidence intervals were broad due to the low frequency of children without language disorders in the investigated sample.

**Table 3:** Logistic regression (with frequencies) and odds ratio calculated for each of the six measures and significant association with diagnostic axes 1 and 2.

			%	OR	95% CI		p
					**Lower**	**Upper**	
**A1 – Clinical disorders^a^**	Self-care	Atypical	74.10	1			
		Typical	54.40	0.25	0.02	3.8	0.32
	Drawing	Atypical	90.10	1			
		Typical	55.60	1.31	0.41	4.16	0.65
	Working memory	Atypical	77.80	1			
		Typical	14.54	5.35	1.99	28.95	0.05*
	Visual constructive skills	Atypical	64.80	1			
		Typical	87.30	0.18	0.02	1.6	0.12
	Attention	Atypical	50.00	1			
		Typical	72.70	1.19	0.18	8.01	0.86
	Language	Atypical	55.60	1			
		Typical	3.60	26.78	1.87	383.93	0.02*
A2 – Intellectual disability^b^	Measures						
	Self-Care	Atypical	43.10	1			
		Typical	56.90	20.76	0.49	875.76	0.11
	Drawing	Atypical	87.70	1			
		Typical	18.28	4.8	1.9	25.73	0.05*
	Working memory	Atypical	25.00	3.8	1.6	4.9	0.01*
		Typical	95	1			
	Visual constructive skills	Atypical	90.80	1			
		Typical	54.50	0.23	0.05	1.15	0.07
	Attention	Atypical	76.90	1			
		Typical	61.40	0.52	0.1	2.79	0.44
	Language	Atypical	92.30	1.74	1.02	4.06	0.04*
		Typical	52.30	1			

a Hit rate: 62% - canonical correlation 91%;

b Hit rate: 79.8% - canonical correlation 95%;

* Significant differences at level p<0.05.

On Axis 2, low performance on the drawing and working memory tasks were found to be predictive of positive diagnosis for intellectual deficiency. Statistical analysis showed that the children with drawing problems (87.7%) were 4.8 times more likely to present positive diagnosis on Axis 2 in comparison to children with no problems on this task (18.28%). On the other hand, good performance on working memory can be considered a protective factor for problems on Axis 2. Children with working memory problems (95%) were found to be 4 times more likely to present positive diagnosis on Axis 2 in comparison to children who did not have working memory problems (25.4%). However, children with language problems were 1.74 times more likely to present positive diagnosis on Axis 2.

Considering each model individually gives a correct answer rate of 79.8% (Axis 2) to 84.4% (Axis 1) for logistic models compared to real data. The Cox & Snell index of logistic model adequacy for Axes 1 and 2 was 0.25 (Axis 1) and 0.31 (Axis 2), indicating a low proportion of variance explained by the model. This means that the model created cannot be utilised directly due to its reduced capacity to explain real data. It is noteworthy however that this neuropsychological screening model is composed of sensitive attributes for the detection of positive diagnosis on Axes 1 and 2 of DSM-IV, rendering it a valid preliminary diagnostic procedure for learning difficulties, with consistent application for the identification of children with higher risk of specific disorders.

Thus, the neuropsychological screening model was analysed in relation to its diagnostic capacity, based on the statistical analyses described. The researchers propose that this screening model be applied by duly trained health or education professionals, requiring an application time of approximately 1.5 hours, as the first stage in the screening of children with learning difficulties in case of early referral for a more conventional and in-depth diagnostic investigation. Table 4 provides a comparison of aspects of the procedures as well as the execution time of the interdisciplinary assessment model versus the qualitative neuropsychological screening.

**Table 4:** Comparison of procedures and durations of interdisciplinary assessment model and qualitative neuropsychological screening.

Interdisciplinary assessment	Neuropsychological screening
Anamnesis: 30 minutesProfessional required: pediatrician	Anamnesis: 30 minutesProfessional required: health professional
Pediatric examination: anthropometric measurements and clinical testing (20 minutes)	Qualitative neuropsychological screening (30 minutes) (Appendix 1)
Genetic examination: Presence of dysmorphisms; phenotypes (20 minutes)	Low performance on >4 tasks - referral for neurological and neuropsychological assessment (10 minutes)
Intellectual assessment: Raven; estimated IQ (20 minutes)Professional required: psychologist	Clinical report (20 minutes)
Neuropsychological evaluation: qualitative neuropsychological screening (30 minutes) (Appendix 1) Professional required: interdisciplinary team	---
Tests scores - neuropsychological and behavioral evaluations (30 minutes)Professional required: member of interdisciplinary team	---
Convening of multidisciplinary teams to establish diagnosis (15 minutes)	---
Clinical report (30 minutes)Professional required: member of interdisciplinary team	---
Duration: 3 hours and 30 minutes	Duration: 1 hour and 30 minutes
Number of professionals required: 7	Number of professionals required: 1 (previously trained health professional)

## DISCUSSION

The main objective of this article was to describe a model of a diagnostic investigation conducted among children with learning difficulties, referred by their teachers to a specialised service dealing with neurodevelopmental disorders, based on an interdisciplinary diagnostic investigation especially developed for these children. The diagnostic model included clinical genetic testing, behavioural assessment and screening of neuropsychological functions, in conjunction with conventional cognitive measures - estimated IQ and Raven's Colored Matrices. Although screening measures and measures with qualitative characteristics have less specificity compared to complete neuropsychological tests, our results indicated that these procedures are a valuable tool for intervention in terms of providing fast early detection, cost and accessibility.^6^ Screening tests may have low specificity, raising the need for analysis of incongruence and internal coherence level between variables, which are important for accurate diagnosis. The same holds regarding the use of estimated measures for global intellectual performance, as well as scales that prioritise only one cognitive domain (as occurs with Raven's test, which focuses on non-verbal cognition). The model adopted in these cases, however, allowed the observation of some neuropsychological variables which proved sensitive for the detection of diagnoses on Axes 1 and 2 of DSM-IV, including conduct and oppositional defiant disorders, invasive developmental disorders and specific learning disabilities, such as dyslexia. Some recommendations for the use of this model are therefore necessary.

The results of the statistical analysis pointed to the importance of the neuropsychological assessment screening being applied in its entirety, as the performance variables identified as significant were not sufficiently predictive of diagnosis on Axes 1 and 2. This means that although it might be possible to consider the use of the cited neuropsychological variables alone (working memory and language), found to be sensitive for the diagnosis on Axis 1, the percentage of correct answers in this case was only 62%. When all the tasks were applied and considered as a group in the multivariable approach (canonical correlation), the percentage of right answers increased to 91%. For the diagnoses on Axis 2, when taking into consideration only the 3 significant variables (drawings, working memory and language), the percentage was 79.8%. On the other hand, the application of all procedures resulted in a percentage of right answers above 95% for the same Axis.

We observed a percentage of right answers for diagnoses on Axes 1 and 2 in about 75% when a low performance was identified in five of the adopted tasks; 60% when identified in four tasks; 45% when identified in three tasks; 30% when identified in two tasks; and 15% when identified in only one task. Thus, considering the performance on one task only would increase the risk of error in the diagnosis of 85% of the cases. Consequently, the recommendation for the identification of children with a history of learning difficulties in need of referral for a more comprehensive neurological and neuropsychological assessment, based on the adopted model, would be that of identifying a low performance on a minimum of 4 out the 6 adopted tasks.

The findings concerning the sensitivity of the neuropsychological screening model for diagnoses on Axes 1 and 2 have theoretical support from the literature on Cognitive Neuropsychology. Working memory is a short-term memory system, which involves temporary maintenance and mental manipulation of information, either of verbal or visual spatial nature, and is highly associated with attention and executive functions.^17^ Verbal working memory, for instance, is involved in reading and writing skills and its dysfunctions are evidenced in dyslexia.^4,18-21^ The relationship between working memory and academic performance has also been noted.^22^ The importance of language, in turn, can be analysed if taking into consideration both its communication and instrumental functions in relation to thought and cognition organisation, as sustained by the socio-historical conceptions of development.^23^ Finally, drawing involves an integration of visual constructive and praxical functions, and is also regulated by language and thought.^24^ Together, these three cognitive skills or functions showed a higher rate of right answers for diagnosis on Axis 2 - which concerns the presence of intellectual deficiency - in comparison to Axis 1.

One final consideration concerns the recorded observations on Axes 3 and 4. Medical conditions associated with the main diagnoses identified in cases participating in this study included, for instance, malnutrition, epilepsy and dental occlusion disorders, among others, which imply a demand for differentiated medical referrals. The presence of problems associated with Axis 4 was identified in 41.28% of the families. These problems included, for instance, parents' alcoholism (detected in 23% of cases) and exposure to domestic violence. Although logistic regression analyses did not identify a direct association between socio-environmental variables and diagnoses on Axes 1 and 2, previous studies suggest that the impact these factors may have on children's development should not be underestimated.^25,26^ It has been claimed, for instance, that environmental factors such as low income, families with a high number of children, single parenting, maternal depression, paternal absence, low parental educational level or psychiatric problems are as relevant as biological risk factors, such as low birth weight and malnutrition, for the development and mental health of children. Evidence indicates that favourable socio-demographic conditions and quality of environmental stimulation are associated with a higher level of social competence as well as lower rates of psychiatric morbidity.^27,28^ As a result of these aspects, it has been proposed that investigations into family structure and dynamics become an increasingly present component in clinical assessments.^29^ Hence, the DSM-IV multiaxial system can contribute substantially to differential diagnosis in learning difficulties, and to a more in-depth understanding of cases on an individual basis.

To conclude, this article presented a diagnostic investigation model of an interdisciplinary approach based on qualitative procedures for neuropsychological assessment, which constitutes preliminary actions for differentiation between learning difficulties and disabilities. We also propose the use of neuropsychological screening indistinctively by professionals from several health areas. We are aware of the need to test this screening model in larger samples and to train health and education professionals on its use and on the DSM-IV multiaxial diagnostic system. However, models with such characteristics that can be incorporated into public health services could prove useful for health centers throughout Brazil.

## APPENDIX 1 - NEUROPSYCHOLOGICAL SCREENING

**Table:**

**Identification**				
N°_____ Name: ____________________________________________________________________________________________ Date of birth _____/_____/_____; School grade _________________________				
Mother's name: ________________________________________________________________________________________________ Mother's educational level: __________________________________				
**Clinical conditions**				
Delivery: Normal _____________________________________ / Cesarean _____________________________________ ; Birth weight: ___________________________________________ ; Hospital stay (days) _____________________________________				
Current weight: ___________________________________________________ Current height ____________________________________________________ Cephalic perimeter ______________________________________________________				
Presence of dysmorphisms: minor _________________________________________ major ______________________________________ (number)				

	Skill/ Tasks		**Performance**	**Observations**
**Drawing**	CLASSIFICATION			
	1. Squiggly lines		__________	
	2. Cells		__________	
	3. First figures that arise from cells		__________	
	4. Recognizable and well-structured figures		__________	
	5. Complete scene		__________	
**Visual constructive skills**	COPYING OF SIMPLE SHAPES			
	1. **T**		__________	
	2. **⚪**		__________	
	3. +		__________	
	4. ◊		__________	
**Language**	PRAGMATIC ASPECTS			
	1. Maintain eye contact in communication		__________	
	2. Starts conversation spontaneously or communicates by gestures		__________	
	3. Answers appropriately to what is requested		__________	
	Verbal expression			
	1. Normal speed of speech		__________	
	2. Good articulation skills		__________	
	3. Appropriate temporal sequence of speech		__________	
	4. Vocabulary typical of age		__________	
	5. Appropriate sentence structure		__________	
	VERBAL COMPREHENSION			
	Story comprehension			
	Story: "Two boys were playing ball. One of them kicked the ball high, the ball hit the			
	vase, the vase fell and broke. When their mother arrived, she was very angry."		__________	
	Question: "Why was the mother angry?"		__________	
**Attention**	FINDING FIGURES			
	1. Horse		__________	
	2. Scissors		__________	
	3. Strawberry		__________	
	4. Airplane		__________	
	5. Cup		__________	
	6. Pineapple		__________	
**Working memory**	VERBAL			
	Oral repetition of digits			
	a) Forwards: 2-9-5		__________	
	b) Forwards: 3-1-8-5		__________	
	c) Backwards: 8-1-6		__________	
	d) Backwards: 4-8-3-7		__________	
	VISUAL			
	Pointing at squares			
	a) Forwards: 1-5-3		__________	
	b) Forwards: 2-1-4		__________	
	c) Backwards: 5-3-2-1		__________	
	d) Backwards: 4-3-2-5		__________	
**Self care**	Food		__________	
	Clothing		__________	
	Hygiene		__________	
Intellectual performance:				
- IQ __________; Raven's Colored Matrices: ________ (percentile)				

**Qualitative analysis of performance**				
- Drawing (0) / (1) / (2);				
- Visual Constructive Skills (0) / (1) / (2);				
- Language (0) / (1) / (2);				
- Attention (0) / (1) / (2);				
- Working memory (0) / (1) / (2);				
- Self-care (0) / (1) / (2);				
**Number of well-developed skills (typical for chronological age):_______**				
**Future referrals required**				
- Medical examination: pediatric / genetic				
- Full neuropsychological assessment				
	Examiner: _______________________ Screening date:_____________			
